# CCT: Lightweight compact convolutional transformer for lung disease CT image classification

**DOI:** 10.3389/fphys.2022.1066999

**Published:** 2022-11-04

**Authors:** Weiwei Sun, Yu Pang, Guo Zhang

**Affiliations:** ^1^ College of Optoelectronic Engineering, Chongqing University of Posts and Telecommunications, Chongqing, China; ^2^ School of Medical Information and Engineering, Southwest Medical University, Luzhou, China

**Keywords:** axial attention, compact convolutional transformer, COVID-19, positional bias term, image classification

## Abstract

Computed tomography (CT) imaging results are an important criterion for the diagnosis of lung disease. CT images can clearly show the characteristics of lung lesions. Early and accurate detection of lung diseases helps clinicians to improve patient care effectively. Therefore, in this study, we used a lightweight compact convolutional transformer (CCT) to build a prediction model for lung disease classification using chest CT images. We added a position offset term and changed the attention mechanism of the transformer encoder to an axial attention mechanism module. As a result, the classification performance of the model was improved in terms of height and width. We show that the model effectively classifies COVID-19, community pneumonia, and normal conditions on the CC-CCII dataset. The proposed model outperforms other comparable models in the test set, achieving an accuracy of 98.5% and a sensitivity of 98.6%. The results show that our method achieves a larger field of perception on CT images, which positively affects the classification of CT images. Thus, the method can provide adequate assistance to clinicians.

## 1 Introduction

According to real-time statistics from the World Health Organization (WHO) and Hopkins University, as of 1 August 2022, there were an estimated 570 million confirmed COVID-19 cases worldwide, with −6.4 million deaths (Dong et al., 2020; Zhu et al., 2020). With an increasing number of new cases recorded worldwide, COVID-19 has considerably impacted industries. Additionally, people’s everyday lives have been seriously affected. Therefore, the primary means of prevention and detection entail controlling the spread of COVID-19. In clinical settings, nasopharyngeal and oropharyngeal swabs are the main screening methods for COVID-19 (Xu et al., 2020). However, many circumstances might cause a false negative test result (Bai et al., 2020). For example, at the initial stage, when the virus enters the human body, the amount of virus present in the human body is within an undetectable level. And different sampling times and locations may yield insufficient viral amounts in the samples. In addition, the laboratory equipment and the testing capabilities are poor, and a quality management system has not been established. Thus, these restrictions increase the risk of COVID-19 transmission and cause patients to receive delayed treatment or a wrong diagnosis.

The advantages of computed tomography (CT) are noninvasiveness, high resolution, and timeliness, which help diagnose COVID-19. CT expedites the diagnostic processes and is an effective supplement to nucleic acid detection. CT images can clearly find lesions, observe their size, shape, texture, and other characteristics, and accurately segment them (Bernheim et al., 2020; Rubin et al., 2020; Wong et al., 2020). Analyzing the degree of pulmonary involvement and the severity of infection helps support the follow-up clinical treatment of patients. However, community pneumonia (CP) is also associated with cough, sputum, malaise, and fever (Afshar et al., 2020; Zhang et al., 2020a; Brunese et al., 2020; Han et al., 2020; Mahmud et al., 2020; Oh et al., 2020; Ozturk et al., 2020; Calderon-Ramirez et al., 2021; Ozyurt et al., 2021), and CT images of community pneumonia are very similar to COVID-19. This not only makes it more difficult to read the images (Shi et al., 2020) but also greatly increases the workload of the doctors. Further, manually labeling the infected area is time-consuming, and the accuracy is subject to the doctor’s subjectivity.

Deep learning (Li et al., 2009; Li et al. 2010; Li et al. 2015; Ardakani et al., 2020) has demonstrated excellent capabilities in auxiliary lung diagnosis recently. It can automatically mine high-dimensional features related to clinical outcomes from CT images. The deep learning-based COVID-19 image classification model has successfully assisted in patient disease diagnosis (Esteva et al., 2017; Litjens et al., 2017; Ardila et al., 2019; Esteva et al., 2019; Qin et al., 2019; Topol 2019; Mei et al., 2020; Sun et al., 2022). An automatic and accurate method for COVID-19 detection based on the ResNet50 model was proposed (Li et al., 2020). And 4,356 chest CT images of 3,322 patients were used to distinguish between COVID-19, CP, and non-pneumonia. The sensitivity, specificity, and area under the curve (AUC) scores of the model were 90%, 96%, and 0.96, respectively. A method for COVID-19 detection based on the DenseNet201 depth transfer model was proposed (Jaiswal et al., 2020). The model was trained using the Image Net dataset and was 96.3% accurate in classifying and recognizing chest CT images. Further, Wu et al. integrated COVID-19 classification and lesion segmentation into the COVID-CS network, and the two tasks shared the same backbone network (Wu et al., 2021). The classification test set obtained an average sensitivity and specificity of 95.0% and 93.0%, respectively. Some researchers built Dense Net-121 to identify the CT images of COVID-19 in a comparative experiment to achieve self-supervised learning and an accuracy of 85.5% (Chen et al., 2021).

However, the classification of COVID-19 still has the following problems. At present, many algorithms (Li et al., 2020; Wang et al., 2020; Hassani et al., 2021) can be used to partially solve the problem of scarce COVID-19 data. But most methods are difficult to accurately capture the essential feature space of various categories of data in a small amount of image data. And, most of the existing algorithms have poor classification performance for common pneumonia and COVID-19, which seriously affects the overall classification performance of the algorithms. It will hurt the subsequent research and eventually make the algorithms difficult to be applied in the clinic.

Therefore, to increase the recognition ability of the model for common pneumonia and COVID-19, and further improve the accuracy and efficiency of COVID-19 image recognition, we employ a novel method to solve the above problems in the CT image classification of COVID-19. A new sequence pooling approach and convolution are proposed herein, i.e., a smaller and more compact transformer based on CCT suitable for datasets lacking pneumonia images. First, the self-attention mechanism in CCT is decomposed into two one-dimensional (1D) self-attention mechanisms: height axis and width axis (Ho et al., 2019; Huang et al., 2019). Subsequently, while the axial attention mechanism replaces the original self-attention mechanism, location coding is added to obtain a larger receptive field. Finally, the position offset item is added to the position-coding to obtain the dependence of the precise position information during training. Herein, the addition of the axial attention mechanism considerably improved the accuracy of COVID-19 detection on chest CT images, achieving better performance results for both COVID-19 model accuracy and other pneumonia screenings. The main innovations herein are as follows. 1. A new sequence pooling strategy and convolution are proposed along with a smaller and more compact transformer based on CCT; this transformer is suitable for datasets lacking pneumonia images. 2. We improved the self-attention mechanism of the transformer encoder to an axial attention mechanism and added a position offset term. The long-range location dependencies of accurate location information are obtained during training to improve the model’s classification performance. 3. Compared to the Vision Transformer (ViT) structure and the traditional Convolutional Neural Network (CNN), the performance on the small COVID-19 dataset is stronger.

## 2 Materials and methods

**FIGURE 1:**
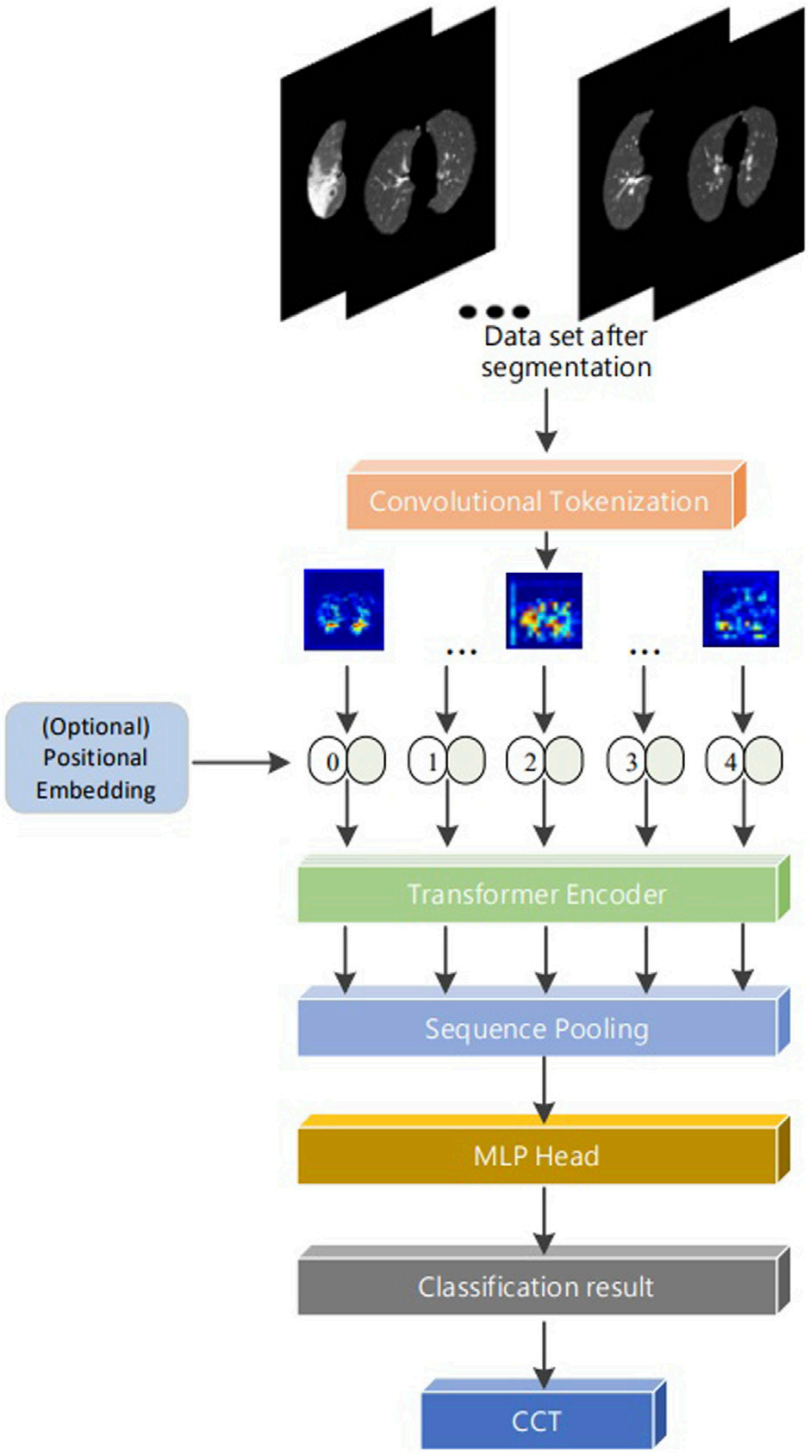
Our compact convolutional transformer models.

Our proposed sequence pooling method and convolution module of the CCT model can reduce the class token and embedding requirements. The convolution module can be adapted to the small COVID-19 dataset. The model belongs to the lightweight transformer structure and comprises a convolution module, embedding, transformer encoder, sequence pooling, and multilayer perceptron (MLP) (Ramchoun et al., 2017) head (Figure 1).

### 2.1 Improved compact convolutional transformer model structure

**FIGURE 2:**
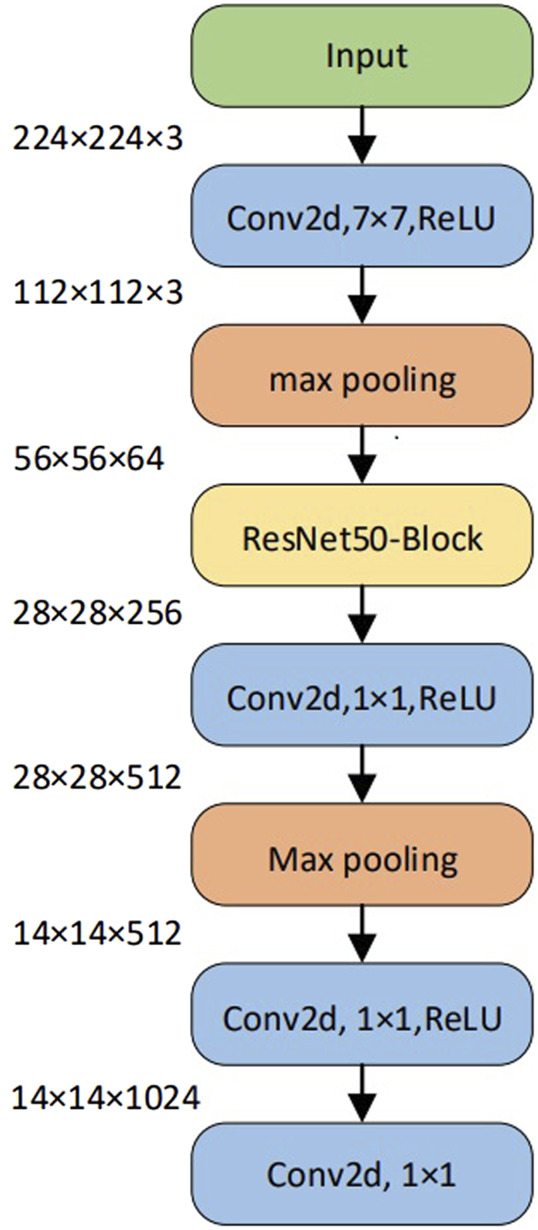
CCT diagram of convolution module.

We propose a patching method based on small-scale convolutional modules in the CCT to completely preserve local information. This method does not affect how the transformer encoder calculates patch interactions. First, after the input feature vector of the convolution module is normalized, the convolution operation and the ReLU function are used for feature extraction. Second, down-sampling through max-pooling extracts essential information. Third, the residual structure of ResNet50 is employed as an additional feature extraction to prevent the transformer structure data from being unable to be trained during the backpropagation process. Finally, the output vector processed by the convolution module meets the input dimension requirements of the embedding layer. Subsequently, the 3D vector is down-sampled, and the ReLU activation layer is performed. After convolution and flattening operations, the vector dimension of the same size as a position embedding layer of the improved model is obtained (Figure 2).

The CCT can adapt to training with smaller datasets by adjusting the size of patches. The CCT introduces a patching method based on convolution. The relationship between patches can be encoded while restraining the local information. This method can effectively tokenize and maintain the local spatial relationship, thereby eliminating dependence on the class token and providing greater model flexibility.

### 2.2 Transformer encoder

**FIGURE 3:**
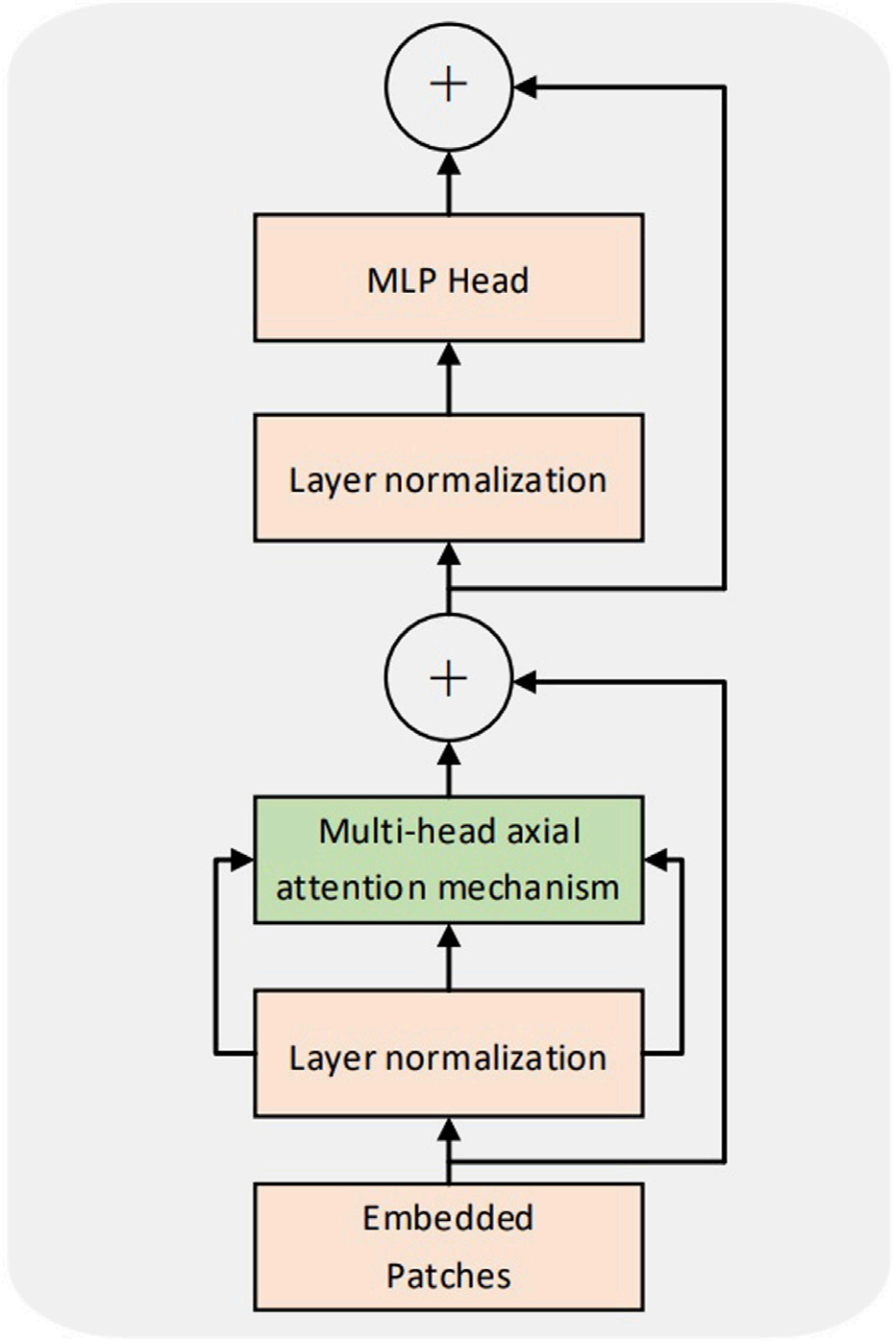
Encoder structure drawing.

The transformer encoder of the CCT was consistent with that of the ViT. Multiple encoders exist in the model, with no weight sharing among them. Figure 3 illustrates the structure of the encoder. Each coding layer comprises two sublayers: multihead self-attention (MSA) and MLP. Each sublayer is preceded by layer normalization. The input sequence was set to x; the output y of a single coding layer was obtained. The formula is as follows:

$$x_{l-1}^{\prime }=x_{l-1}+W-MSA\left(\mathrm{LN}\left(x_{l-1}\right)\right)$$ (1)

ℓ∈1,2,…,L (2)

$$x_{\ell }=x_{\ell }^{\prime }+MLP\left(\mathrm{LN}\left(x_{\ell -1}\right)\right)$$ (3)

In Eqs 2, 3, a structure similar to the residual network (He et al., 2016) is laid out. This design retains more information, reduces information loss, and can use a more significant number of encoders for training. L denotes the number of encoders.

The transformer can establish distance dependence on the target while extracting more powerful features by multifocusing on the global content information. The self-attention mechanism in the encoder, given a height of h , a width of *w*, and a channel of input embedded patches $X\in R^{h\times w\times c_{in}}$ , and an output formula $y_{o}\in R^{h\times w\times c_{out}}$ with position o={i,j∣i∈{1,…,h},j∈{1,…,w}} is defined as follows:

$$q=W_{Q}x$$ (4)

$$k=W_{K}x$$ (5)

$$v=W_{V}x$$ (6)

$$y_{o}=\underset{p}{\sum }soft\max\left(q_{o}^{T}k_{p}\right)v_{p}$$ (7)

The *q*, *k*, and *v* vectors in Eqs 4–6 are the query, key, and value, respectively. W_Q_, W_K_ and $W_{V}\in R^{\left(c_{in}\times c_{outn}\right)}$ are the weight matrices learned during training. In Eq. 6, v is multiplied by the input xi and the trainable matrix WV to obtain the input eigenvector. The dot product of q and k is used to calculate the weight of *v*. In Eq. 7, p=(w, h) , q and k are normalized by SoftMax and multiplied by v to obtain the attention value. In contrast to convolution, the self-attention mechanism may obtain nonlocal information from the entire feature map. However, the calculation of this attention value comes at a cost. Applying the self-attention mechanism to the visual model architecture becomes impossible as the feature map increases. Additionally, the self-attention layer does not use any position information when calculating the nonlocal context. However, the position information is vital for obtaining the structure and shape of the target in the visual model.

**FIGURE 4:**
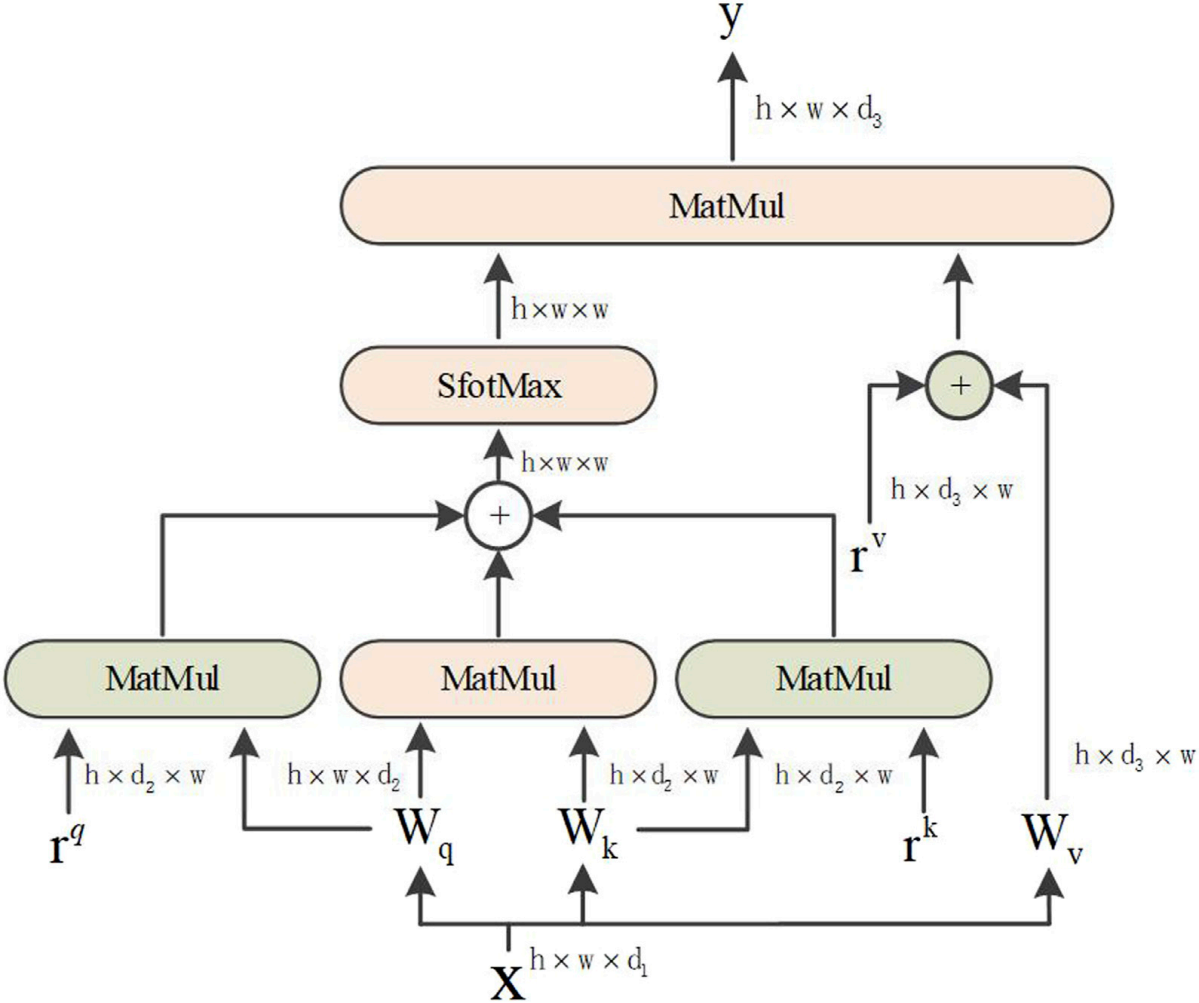
Axial attention.

Based on the abovementioned reasons, the axial attention mechanism is divided into two 1D self-attention mechanisms: the height and width axes. Additionally, a position code was added to the query mechanism. The structural diagram is shown in Figure 4. The axial attention mechanism can also match the original self-attention mechanism dimensions. The width and height dimensions are considered to reduce the number of calculations and improve the calculation efficiency. The position offset terms are set while collating the attention value to make it more responsive to the position information. This bias term is usually called relative position coding and can be learned through training.

The attention model of Ramachandran et al. uses relative position coding for queries only. This study combines the axial attention mechanism and position coding to apply them to all queries, keys, and values. For any given input feature map *x*, an axial position-sensitive attention mechanism with position encoding along the width-axis, the equation is as follows:

$$y_{ij}=\overset{p=1}{\underset{p=1}{\sum }}soft\max\left(q_{ij}^{T}k_{iw}+q_{ij}^{T}r_{iw}^{q}+k_{ij}^{T}r_{iw}^{k}\right)\left(v_{iw}+r_{iw}^{v}\right)$$ (8)

where $r^{q},r^{k},r^{v}\in R^{W\times W}$ , $r_{iw}^{q}$ , $r_{iw}^{k}$ , and $r_{iw}^{v}$ are learnable vectors representing the position codes of queries, keys, and values. For example, the attention mechanisms of the height and width axes have the same definition. One axial attention layer spreads information on a specific axis, and both axial attention layers use an MSA mechanism. After the position offset term is an introduction to the axial attention mechanism Compared to the original self-attention mechanism, the global receptive field acquisition feature can be realized, thus reducing the computational complexity.

### 2.3 Serial pooling

Herein, the feature vector classification results are output using sequence pooling rather than class tokens (Devlin et al., 2018). For the L-layer transformer encoder, the output results are collected in sequence. The model is compact as the data sequence includes information and category information for different parts of the input image, thereby compacting the model. Sequence pooling outputs the sequential embedding of the latent space generated by the transformer encoder to correlate the input data better. The output feature mapping is defined as $T:R^{b\times n\times d}\mapsto R^{b\times d}$ , and the equation is given as follows:

$$X_{L}=f\left(X_{0}\right)$$ (9)

where X_L_ or f (X_0_) is the L-layer Transformer encoder, b is the batch size, n is the sequence length, d is the embedding dimension, and $\left(X_{L}\right)\in R^{d\times 1}$ . Using the SoftMax activation function, the equation is given as follows:

$$X_{L}^{\prime }=soft\max\left(g{\left(X_{L}\right)}^{T}\right)$$ (10)

As $\left(X_{L}\right)\in R^{d\times 1}$ , we get:

$$Z=X_{L}^{\prime }X_{L}=soft\max\left(g{\left(X_{L}\right)}^{T}\right)\times X_{L}$$ (11)

where $z\in R^{b\times 1\times d}$ merge the second dimension to get $z\in R^{b\times d}$ . This output can then be used to obtain the result through a linear classifier.

### 2.4 Datasets

#### 2.4.1 Lung data COVID-19 CT-CCII

**FIGURE 5:**
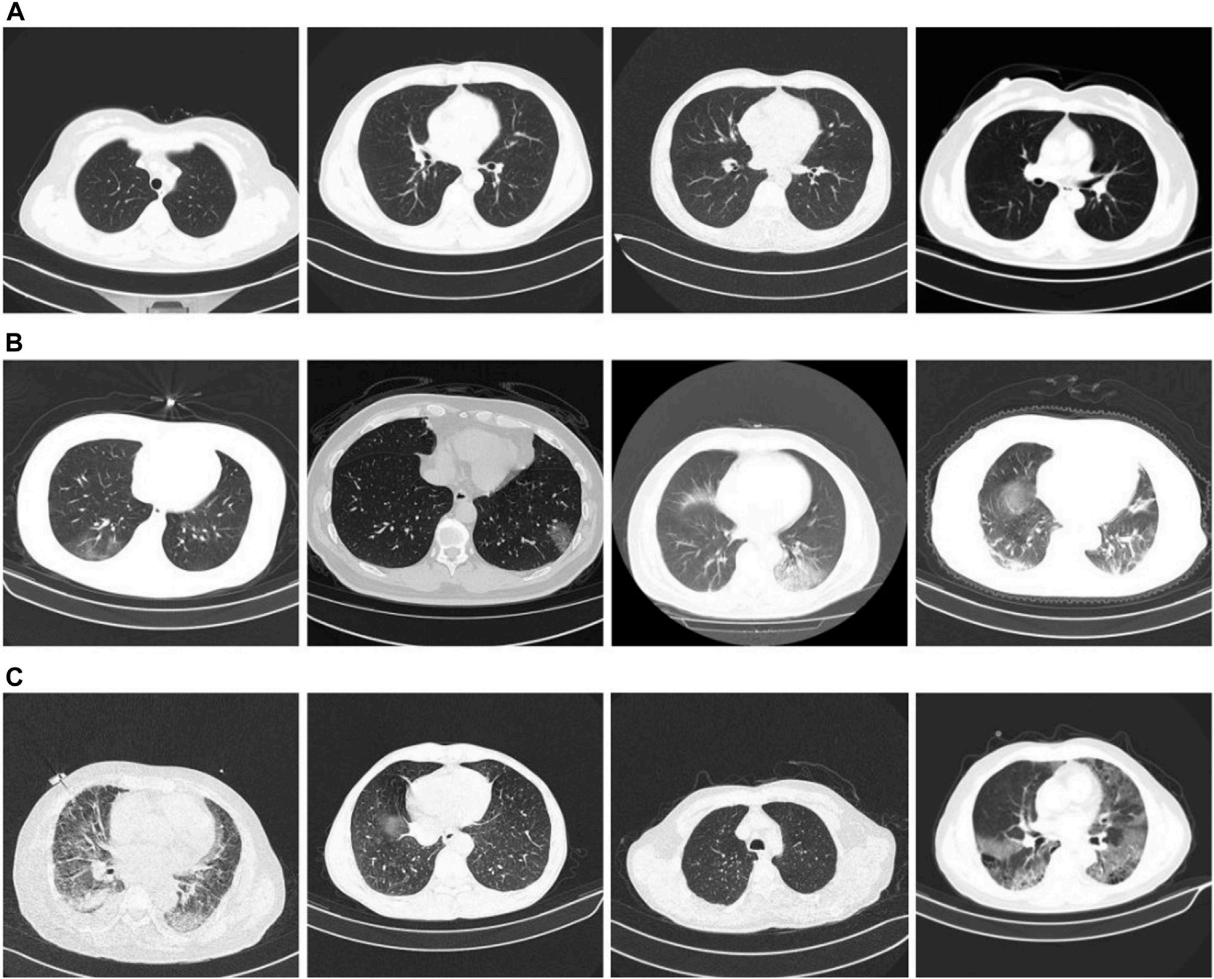
CC-CCII chest CT images. **(A)** Normal conditions; **(B)** COVID-19; **(C)** CP.

We used the classification dataset from the China Consortium for Chest CT Imaging Research (CC-CCII) (Zhang et al., 2020b; Zhou et al., 2021). Informed consent from the patients was obtained, reviewed, and approved by the Medical Ethics Committee. The dataset comprises 6752 CT scans of 4,154 participants. For our training test, we used 5985 CT scans. Among them, the training set is 3,017, and the test set is 2,968. The training and test set distributions were consistent, and the ratio of COVID-19, community pneumonia, and normal in the dataset was 1:1:1. The image size is three-channel, 512 × 512 × 3. Figure 5 presents an example of the dataset. Figures 5A–C show CT images of a typical patient, a patient with COVID-19, a patient with community pneumonia (mainly bacteria, viruses, chlamydia, and other microorganisms causing pneumonia), respectively.

#### 2.4.2 Dataset partitioning

To divide the dataset, the K-Fold cross-validation method was employed. First, the dataset was divided into K sets, and each fold training used K-1 sets as the training set to train the model (K = 10). The remaining set was used as a validation set to test the performance evaluation of each folded training model, and the content of each validation set remained unrepeated. The data augmentation methods of random rotation, horizontal flipping, and contrast adjustment were used in training pre-processing to improve the model’s generalization ability.

#### 2.4.3 Experimental environment

Ubuntu18.04 was used as the operating system platform, with Intel(R) Core (TM) i5-6500 CPU, Nvidia GeForce GTX 1080ti GPU, with 11 GB of video memory and 16 GB of RAM.

The model performance can be improved, and the training time can be reduced with proper parameter configuration. Stochastic gradient descent was used to train the optimizer, and exponential decay was used to adjust the learning rate. The initial learning rate is 0.001. Additionally, 10-fold cross-validation was used for training with 100 epochs per fold. The details of the experimental training parameters are shown in Table 1.

**TABLE 1:** Training parameter settings.

Type	Setting
Batch size	16
Learning rate	0.001
Optimizer	SGD
Epoch	100
Ubuntu 18.04	PyToch1.6.0

#### 2.4.4 Evaluation indicators

To analyze the classification performance of the trained model for COVID-19, CP, and normal, three performance metrics were used: accuracy (Acc), sensitivity (Sens), and AUC of the receiver operating characteristic (ROC). The mPA is the average sum of each category’s pixel accuracies. The formulas are as follows:

$$Acc=\frac{TP+TN}{TP+FN+FP+TN}$$ (12)

$$Sens=\frac{TP}{TP+FN}$$ (13)

$$mPA=\frac{1}{k+1}\overset{k}{\underset{i=0}{\sum }}\frac{TP}{TP+FN}$$ (14)

where, TP represents the number of positive examples that are predicted to be positive examples; FP represents the number of negatives predicted as positives; FN represents the number of positive classes predicted to be negative; TN represents the number of negative classes predicted to be negative; k is the number of categories.

Assuming that the ROC curve is formed by continuous links of points whose coordinates are $\left\{\left(x_{1},y_{1}\right),\left(x_{1},y_{2}\right),\ldots ,\left(x_{m},y_{m}\right)\right\}$ . The AUC formula is as follows:

$$AUC=\frac{1}{2}\overset{m-1}{\underset{i=1}{\sum }}\left(x_{i+1}-x_{i}\right)\times \left(y_{i}+y_{i+1}\right)$$ (15)

FLOPs=(2×I−1)×O (16)

where, where *I* and *O* represent the input and output neuron numbers, respectively.

## 3 Results

### 3.1 Ablation experiment

**FIGURE 6:**
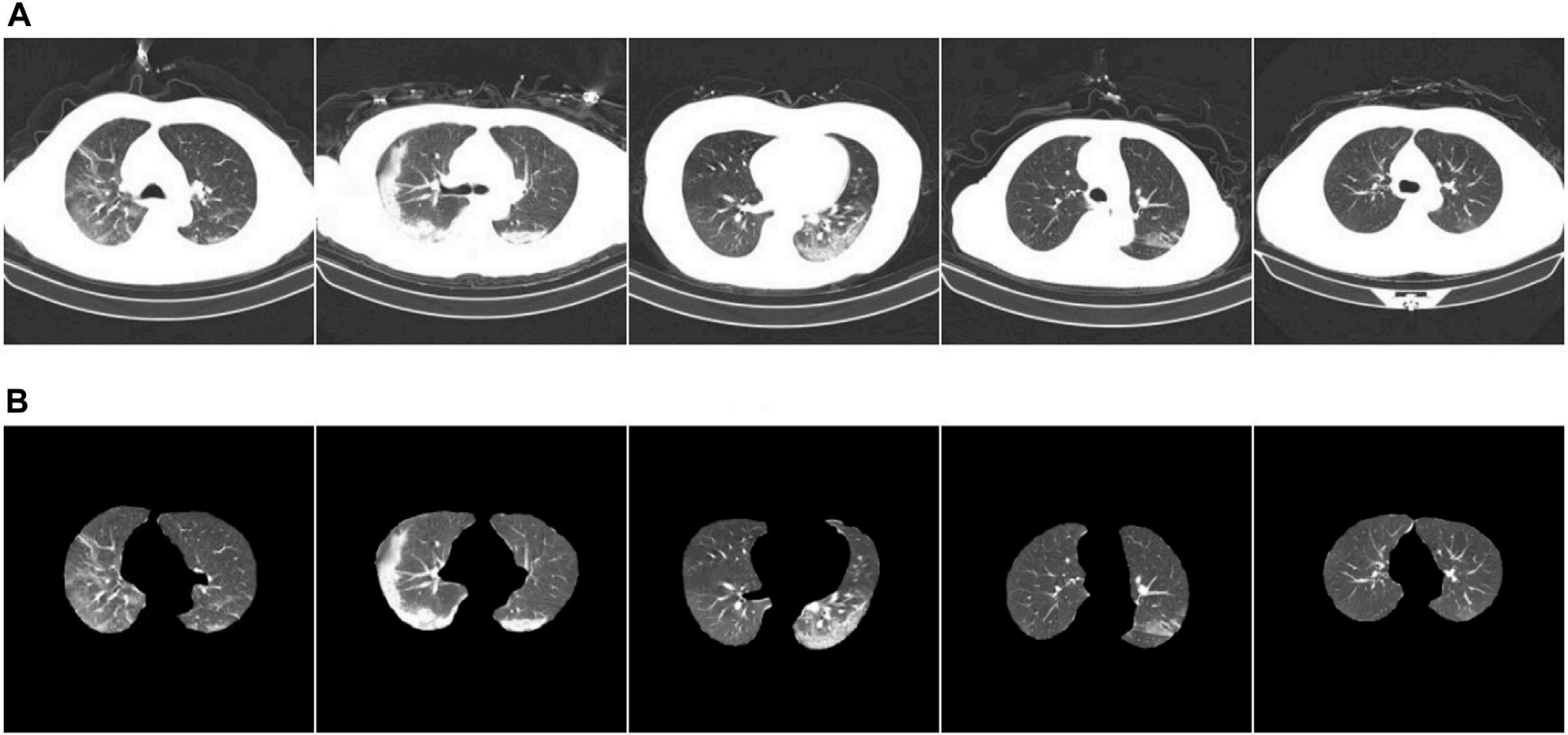
Example of ablation experiment comparison. **(A)** The CT image of the COVID-19 patient; **(B)** the result.

COVID-19 pneumonia and other pneumonia lesions exhibit the same characteristics of being in the lung area. However, the chest CT scans contain other interfering areas. To ensure that the lung area was unaffected by the interference area, preprocessing was performed during classification training to segment the lung area from the chest CT image. Next, ablation experiments were conducted to verify the segmentation effect of the new, improved model. The segmentation test results are presented in Figure 6. Figure 6A shows the CT images before segmentation, and Figure 6B shows the CT images after segmentation in Figure 6A from left to right. The results show that the newly proposed method can segment tiny lesion details, achieving the highest segmentation performance.

We compared our model to other models to more accurately evaluate its performance. The results are presented in Table 2. First, the convolutional neural network was used to extract enough local information after preprocessing the image features through the convolution module during input. Next, the vector was input into the improved transformer structure, and the initial self-attention mechanism was replaced with the axial attention mechanism. Further, a position offset term was added to improve the model performance. Compared with the CCT model, the accuracy and sensitivity of our improved model are increased by 1.7% and 2.3%, respectively, and the number of floating-point operations (FLOPs) is less than the model calculation amount of the CCT model. Concerning the recognition speed of a single image, the lightweight CCT single image recognition speed is the fastest, only 0.014 s. This is faster than all other models, and its recognition accuracy has not dropped. The comparative results show that our proposed improved method achieves the best results in screening COVID-19 and CP.

**TABLE 2:** Performance comparison of different models.

Modle	Acc/%	Sens/%	AUC	FLOPs (G)	Time (s)
Efficientnet-b7 Tan and le., 2019	88.4	88.3	0.972	1.02	0.023
Mobilenet-v3 Howard et al., 2019	97.8	97.6	0.997	0.33	0.019
ViT (Nielsen et al., 2015)	95.7	95.6	0.992	0.73	0.017
CCT (Esteva et al., 2019)	96.8	96.3	0.993	1.03	0.015
Ours	98.5	98.6	0.999	0.91	0.014

**FIGURE 7:**
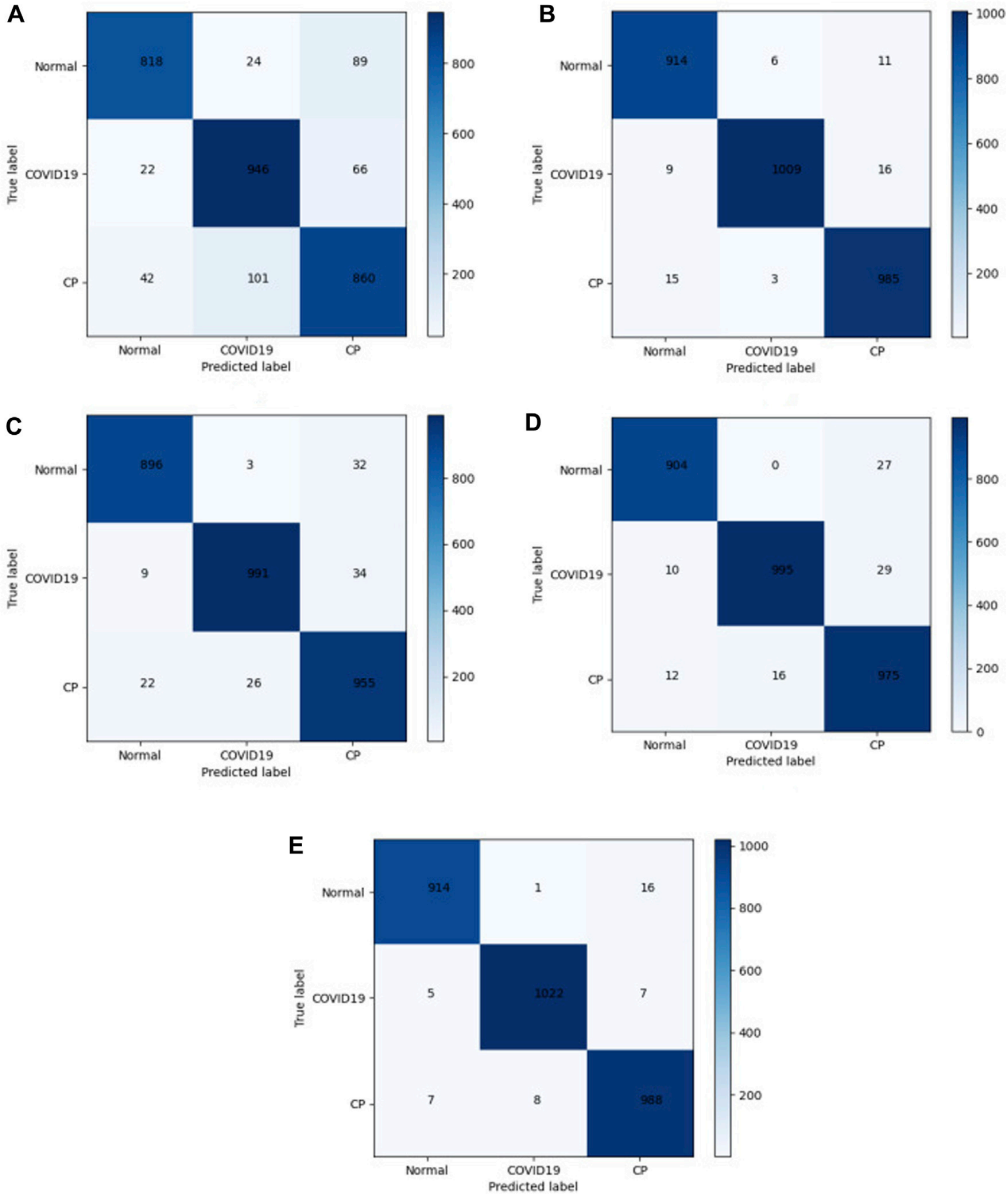
Confusion matrix. **(A)** Efficientnet-b7; **(B)** Mobilenet-v3; **(C)** ViT; **(D)** CCT; **(E)** Ours.

In the medical image application of the transformer, the input patch size parameter setting affects the model performance. The self-attention mechanism in the transformer structure has the advantage of obtaining global contextual connections. The matrices of different models were used to evaluate the performance. A total of 2968 CT images were tested. The confusion matrix in Figure 7 shows the difference between the actual and predicted values. The horizontal axis represents the model prediction results, corresponding to the number of predictions of different categories. The vertical axis represents the ground-truth labels (normal, COVID-19, and CP). A 3 × 3 matrix was used to compute the TP, FP, and FN values of the multiclassification task. The numbers on the blue back-ground are the number of correct predictions by the model. The values in the other regions correspond to the values at which the model predicted incorrectly, and the confusion matrix clearly shows the number of types of model mispredictions. The results show that the discrepancies between the chest CT images taken under normal conditions and during pneumonia have different presentation characteristics, leading to differences. Thus, it is easier to make sound judgments about the model. However, a small number of patients with mild COVID-19 or CP are mistaken for normal owing to a lack of apparent symptoms on chest CT images. Each model showed varying degrees of misidentification, misidentifying both COVID-19 and CP as usual. This misidentification is due to certain similarities between chest CT images of COVID-19 patients and other pneumonia patients, such as ground-glass opacity and lung parenchyma features. Among them, Mobilenet-v3 and our model have fewer misidentifications. The test results in Figure 7E show 1034 COVID-19 CT images. Five and seven CT images were wrongly identified as normal and CT, respectively. The misdiagnosis rate is lower than in other compared models. Our improved model achieved the highest accuracy and the lowest misdiagnosis rate.

### 3.2 Real dataset model performance comparison

(1) COVID-CT dataset

We investigated the performance of different models on the COVID-CT dataset. Yang et al. collected 349 COVID-19 and 397 normal chest CT images in the COVID-CT dataset for 216 patients (Yang et al., 2020). However, some image data in this dataset were marked or missing. Image quality may have some impact on the training of the model. He et al., 2020 used contrastive self-supervised learning for training and achieved a model accuracy of 86%. Shalbaf et al. used 15 CNN benchmark models for fine-tuning training with the best accuracy and sensitivity of 84.7% and 82.2%, respectively (Gifani et al., 2021). Table 3 shows the comparison between our method and the methods above. The findings demonstrate that their training programs have engaged in significant workloads and relatively complex data preprocessing. However, our improved method achieves the best performance results in the COVID-CT dataset.

(2) SARS-CoV-2 CT-scan dataset

**TABLE 3:** Model comparison of COVID-19 CT dataset.

Model	Acc/%	Sens/%
He et al., 2020	86	—
Gifani et al., 2021	84.7	82.2
Ours	87.3	86.7

The SARS-CoV-2 CT-scan dataset comprises 2,482 chest CT images, including 1252 COVID-19 and 1,230 non-COVID-19 CT images. Soares et al. proposed the xDNN model and divided the dataset into training and test sets in a 4:1 ratio (Soares et al., 2020). After training, the accuracy and sensitivity rates of the model were 97.38% and 95.53%, respectively, and the essential auxiliary diagnosis ability was realized. Panwar et al. proposed an improved VGG model and used the dataset for training and testing, and the final sensitivity was 94.04% (Panwar et al., 2020). The comparison results between our method and the above methods are presented in Table 4. The results show that our improved method achieves the best performance results, with accuracy and sensitivity values of 98.01% and 98.23%, respectively.

**TABLE 4:** Model comparison of SARS-CoV-2 CT-scan dataset.

Model	Acc/%	Sens/%	Specificity/%
Soares et al., 2020	97.38	95.53	—
Panwar et al., (2020)	—	94.04	95.86
Ours	98.01	98.23	98.62

### 3.3 Subjective evaluation

The classification performance of our models was assessed using a more specific evaluation. Ten lead physicians with over 5 years of clinical experience in radiology were invited to perform independent image analysis (sharpness, resolution, invariance, and acceptability). The scoring method of subjective evaluation is presented in Table 5. One hundred CT images of the lesion area were randomly selected, and 10 sets of test samples were constructed equally.

**TABLE 5:** Subjective quality evaluation of a scoring method.

Score	Features of the restored image
0	Severely distorted images
1	Images with severe distortion in some areas
2	Slightly distorted images
3	Difficult to spot distorted images
4	Images with better visual effects
5	Very sharp images

The subjective quality evaluation results of clinicians are shown in Table 6. The results show that our proposed lightweight CCT achieves the best subjective quality evaluations regarding sharpness, resolution, invariance, and acceptability. This is thanks to our improved ViT as a network framework, using an attention mechanism to compute from image height and width separately, adding a position offset term to improve the model classification performance, and our proposed method has the best performance in maintaining edge and texture feature classification.

**TABLE 6:** Subjective quality evaluation of different algorithms.

Method	Sharpness	Resolution	Invariance	Acceptability
Efficientnet-b7	3.4 ± 0.35	3.6 ± 0.18	0.5 ± 0.41	3.8 ± 0.54
Mobilenet-v3	3.6 ± 0.72	3.9 ± 0.26	0.6 ± 0.55	3.9 ± 0.18
ViT	3.6 ± 0.39	4.1 ± 0.51	0.6 ± 0.89	4.1 ± 0.36
CCT	3.8 ± 0.65	4.2 ± 0.13	0.7 ± 0.21	4.1 ± 0.68
Proposed	3.9 ± 0.74	4.3 ± 0.29	0.7 ± 0.96	4.2 ± 0.71

## 4 Discussion

The automatic classification and recognition of chest CT images were improved by improving the CCT model. The self-attention mechanism of the encoder was enhanced to a position-sensitive axial attention mechanism. Meanwhile, the previous architecture was expanded by adding position offset terms to the self-attention mechanism to improve the classification ability of the ability.

Some interference areas were observed in the lung CT images of the patients. Therefore, to keep the model from becoming infected, when the data from the literature were employed simultaneously, sufficient feature extraction of the model was achieved by horizontal and vertical flipping, small angular rotations, and normalized data amplification. Further, it improved the generalization ability of the model to prevent overfitting.

This study CCT employs a new sequence pooling policy, convolution, and smaller, more compact transformers than ViT. Additionally, it compensates for the lack of medical image datasets by eliminating class tokens and positional embedding requirements. However, when the input dimension is large, the model operation cost increases considerably, and global pooling does not use location information when extracting feature information, possibly leading to information loss. Therefore, the self-attention mechanism in the encoder was improved to an axial attention mechanism. The self-attention mechanism was divided into two 1D self-attention mechanisms, the high and wide axes, which were calculated from the two dimensions of the width and height axis. Consequently, the number of calculations and computational efficiency were improved. Additionally, the position deviation was attached to the query, key, and value; an accurate deviation was used to obtain the position information, ensuring that more spatial structural information could be obtained.

According to the results in Tables 2–4, adding the axial attention mechanism considerably improved the accuracy of COVID-19 detection in chest CT images. In small datasets, the performance was better than that of the standard transformer structural network and comparable to that of the traditional CNN. Although the transformer framework classification model may be suitable for small datasets by changing the patch size or encoder structure, some problems remain. For example, a maximum of three categories of models were trained; however, more categories could be used. As lung CT images of patients with mild COVID-19 symptoms are very similar to normal lung CT images, some of the discriminating errors from the lung CT images of patients with mild symptoms were present when the test set was used to validate the model. Consequently, datasets can be added later to improve the model performance. Although deep learning can represent a predictable information relationship, which has good prospects for medical applications, it is challenging in the context of data differences and other factors in medical images.

## 5 Conclusion

Although transformers are generally considered to be suitable only for large-scale or medium-scale training, this study shows that our proposed lightweight CCT classification recognition model works successfully on small data regimes and outperforms larger convolutional models. The performance obtained using the proposed model on the small COVID-19 dataset outperforms the standard ViT structured network and is comparable to the performance of traditional CNNs with significantly reduced computational cost and memory constraints. Experiments show that adding a position offset term by using the axial attention mechanism as a Transformer encoder to compute from the image height and width, respectively, can effectively improve the model classification performance. Our proposed classification method achieves the best performance with 98.5% accuracy and 98.6% sensitivity. The subjective quality assessment by physicians is optimal proving that our method is more suitable for clinical practice. Future studies can utilize a lightweight, compact method for initial screening and segmentation network to segment focal features of COVID-19 from chest CT images. We wish to implement a user interface system for digital image processing using a GUI. The main contents include the design of histogram grayscale transformation, edge detection, smooth filtering, and threshold segmentation for lung CT The main contents include the design of histogram grayscale transformation, edge detection, smooth filtering, and threshold segmentation for lung CT images.

## Data Availability

The original contributions presented in the study are included in the article/Supplementary Material, further inquiries can be directed to the corresponding author.
